# Employment of Manganese in Anemic and Other Affections

**Published:** 1850-03

**Authors:** 


*Employment of Manganese in Anemic and other affections.* By M. Hannon.—Manganese and iron are almost constantly found united in the same minerals, and can be separated with difficulty. Again, iron is not always efficacious in chlorosis, and fails in curing anemia arising from cancers, from tubercles, from prolonged and abundant suppuration, &c. In these cases, it cannot be the iron that is deficient in the blood, but some other ingredient; and it is probable that iron is united to manganese in the blood; and that cases of anemia, unsuccessfully treated by iron, might be cured by manganese. M. Hannon first tried the effects of this agent on himself. He took at first a grain of the carbonate of manganese daily, increasing the dose to four grains by the end of the first week, and to eight grains by the end of the second. At the end of a fortnight, he experienced symptoms of a plethora; the appetite increased, the pulse became stronger, and the color of the interior of the eyelids was heightened. He then administered manganese to some anemic patients; some of them experienced nausea for two or three days, after which the medicine was tolerated. In a short time, its beneficial effects became manifest in the increase of color, in the fuller and more frequent pulse, in the energetic movements and general improvement of the functions.

The presence of manganese in the blood was discovered by M. Millon, who presented a memoir on the subject to the Academie des Sciences of Paris. His observations have been confirmed by M. Hannon.

[Several illustrative cases are given. The first mentioned is one of extreme chlorosis, in which the patient was sent into country air, and took iron for some time, without benefit. We are told that]

The patient was then directed to take one of the following pills daily before breakfast, and another before dinner: Extract of cinchona, carbonate of manganese, of each a drachm. Mix and divide into four grain pills. After she had used these pills for a fortnight, the cheeks and conjunctiva regained their color, and the swelling of the feet disappeared. The following pills were then ordered. Sulphate of manganese, carbonate of soda, of each a drachm; fresh charcoal, honey, of each a sufficient quantity to make a mass, to be divided into four-grain pills. A fortnight after the employment of this medicine, the bellows-sound had disappeared; the pulsations of the heart were strong and loud; and an energetic impulse was felt on applying the hand. There was no syncope; and the appetite had returned. The dose of the pills was increased; and a month after, menstruation occurred, and the patient became plump, and able to bear much exertion. She digested and slept well—in a word, was cured.

[Another case is that of a young lady affected with phthisis:]

Iron, with opium was prescribed; but it increased the cough, and brought on obstinate constipation. Syrup of the phosphate of manganese was then given, with cod-liver oil; the latter being added rather to prevent the contact of air with the manganese, than from any expectation of its producing good effects. The constipation ceased; and the cough became more bearable, and ceased in a fortnight. The patient then began to recover embonpoint. A month after the knuckles assumed a very remarkable brick-red color, which has continued up to the present time—a period of nearly a year and a half.—This patient took three gros (216 grains) of phosphete of manganese, in doses of three grains daily.

Madame R. was affected with cancer of the uterus.—She complained of remitted pain in the hypogastric region, and suffered much while at stool. In the evening, she was troubled with severe lancinating pains, which often continued through the night. She was excessively weak, and of a yellow hue. She was troubled with palpitation, and a bruit was heard in the carotid. The feet frequently swelled. Syrup of the iodide of manganese was given, with syrup of horseradish, for several months. The pains did not leave her, but the anemic appearance completely disappeared. To calm the pains, opium, with extract of hemlock, was prescribed; and the patient became apparently cured.

Mademoiselle M., aged 14, of a scrofulous constitution, had glandular enlargements in the neck, ulceration of the transparent cornea of the left eye, and caries of the first phalangeal bone of the index finger of the right hand. Being the daughter of a peasant, she had lived exclusively on vegetable food; but was ordered to take meat, and to drink beer. Syrup of the iodide of manganese was given in doses of a spoonful two or three times a day. Under the influence of this, and her improved diet, she became less lean; soon after, the cornea regained its transparency, having been washed with a lotion containing gr. ss. of nitrate of silver to an ounce of distilled water. The suppuration of the carious bone ceased, and the finger was cured.

M. G. B., aged 38, had been treated with mercury for some years for constitutional syphilis. The bones were sound; the skin was affected with all kinds of eruptions; the tongue had long been the seat of an obstinate tumor; and there were syphilitic opthalmia and iritis. Fumigation and iodide of potassium were persevered in for several months, but without effect. Iodide of manganese was then given, with syrup of sarsaparilla; and in a month the patient was completely healed. He was directed to continue the use of the manganese; and, as he has not since applied for relief, it is probable that he has had no relapse.

These cases have been selected from a number of similar ones, and show the efficacy of the new remedy proposed. Manganese has in all cases produced a more rapid effect than iron in cases of simple anemia. In the forms of anemia cited, all the cases had resisted iron, and all yielded to manganese. The other cases are respectively of phthisis, cancer, scrofula, and syphilis;—all inducing almost irremediable cachexia, and all rapidly alleviated by manganese. The effects of the manganese, as observed in one case, (phthisis) are remarkable. Iron seldom produces a similar result; if it improves the state of the blood, it increases the cough; so much so, that many practitioners abstain from its use in phthisical cases. In all the scrofulous cases the iodide of manganese, by its salutary and rapid influence, was proved superior to the iodide of potassium. The persistence of the cures obtained by manganese, in comparison with those produced by iron, is very remarkable; no cases of relapse have been observed by M. Hannon. The quantity required to be taken, in order to produce the desired result, is far from being so great as that of iron.—*London Journal of Medicine, Sep.* 1849, *p.* 871.

